# Assessment of Patterns of Infiltration and Relapse of Patients with Glioblastoma of the Occipital Lobe

**DOI:** 10.3390/brainsci16030329

**Published:** 2026-03-19

**Authors:** Michal Schulenkowski, Chun Khai Loh, Michael Back

**Affiliations:** 1 Central Coast Cancer Centre, Gosford 2250, Australia; michal.schulenkowski@gmail.com (M.S.);; 2 Melbourne Medical School, The University of Melbourne, Melbourne 3053, Australia; 3 Sydney Medical School, The University of Sydney, Sydney 2000, Australia; 4 Northern Sydney Cancer Centre, Sydney 2060, Australia; 5 The Brain Cancer Group, Sydney 2065, Australia

**Keywords:** glioblastoma, occipital lobe, radiotherapy, infiltration patterns, progression patterns

## Abstract

**Highlights:**

**What are the main findings?**

- Occipital lobe glioblastoma frequently demonstrates tumour infiltration beyond the primary gyral subsite into anatomically connected brain regions.
- Progression commonly occurs at distant neuroanatomical sites rather than remaining confined to the original tumour location.

**What are the implications of the main findings?**

- Patterns of spread appear consistent with major white matter pathways connecting occipital and distant brain regions.
- Recognition of these patterns may help inform future radiotherapy target delineation.

**Abstract:**

**Background:** Current target volume delineation protocols for glioblastoma utilise uniform or isotropic expansion around the surgical cavity and residual tumour, without considering specific sites at risk for infiltration. Tumours arising in different neuroanatomical sites may demonstrate distinct patterns of infiltration. This study aims to review the infiltration and progression sites for the occipital lobe glioblastoma to identify sites potentially at risk. **Methods:** Patients with occipital lobe glioblastoma managed according to the EORTC-NCIC protocol were identified through a prospective database. Based on MRI analysis, a qualitative description of sites of tumour infiltration and subsequent progression was performed. These were categorised into neuroanatomical subsites adjacent to the occipital lobe: level 1 related to the origin gyrus; level 2 related to adjacent gyral subsites; and level 3 related to subsites that involved distant regions. Patients could be classified in more than one level where multifocal involvement was present at diagnosis or progression. Spatial patterns were assessed in relation to three major white matter tracts: inferior longitudinal fasciculus, cingulum, and corpus callosum. **Results:** A total of 46 patients were analysed. At diagnosis, 20 patients (43.5%) had medial occipital lobe involvement and 26 (56.5%) had lateral involvement. Level 2 and level 3 infiltration were observed in 33 (71.7%) and 27 (58.7%) patients. Progression occurred in 43 patients (93.5%), with involvement at level 1 in 28%, level 2 in 77%, and level 3 in 98%. Lateral tumours demonstrated proportionately higher progression in the trigone (75% vs. 52.6%) and anterior temporal lobe (50% vs. 15.8%, *p* = 0.026), while medial tumours more frequently involved the splenium (47.3% vs. 16.7%, *p* = 0.046). **Conclusions:** Infiltration and progression of occipital lobe glioblastoma may demonstrate distinct neuroanatomical patterns, with spatial distribution corresponding to major white matter tracts.

## 1. Introduction

Glioblastoma is the most common primary brain tumour in adults, and despite subtle advancements in treatment over the past decade, only marginal improvements in survival have been attained [1]. Glioblastoma exhibits aggressive infiltration and recurrence following surgical resection and adjuvant treatment. The neural connectome, the network of white matter tracts within the brain, has been proposed as a plausible route for tumour spread [1]. This may account for glioblastoma progression distant from the initial tumour site, as these pathways connect anatomically distinct brain regions.

Despite an aggressive multimodal treatment approach, the prognosis for glioblastoma remains poor, with median overall survival (OS) ranging from 14 to 18 months [2,3,4,5]. Since 2005, numerous novel therapeutic agents have been trialled in the adjuvant setting; however, none have demonstrated a survival benefit [2,6,7]. These findings underscore the importance of optimising current treatment protocols and reviewing current paradigms.

The current standard of care for newly diagnosed glioblastoma involves adjuvant fractionated radiotherapy combined with temozolomide based on the EORTC-NCIC protocol [8,9]. Despite advances in our understanding of glioblastoma infiltration, as well as improvements in neuroimaging and radiotherapy techniques, this protocol has remained largely unchanged.

Radiotherapy target volume delineation protocols for glioblastoma are largely based on autopsy studies conducted in the 1980s, which demonstrated microscopic tumour infiltration extending beyond 20 mm from the primary tumour mass [10,11]. These studies predated the era of magnetic resonance imaging (MRI) and other advanced neuroradiological techniques. However, they still continue to form the foundation of standard protocols that apply a uniform 15–20 mm isotropic expansion around the tumour bed [8]. This may be suboptimal given evidence that marginal recurrences remain uncommon in protocols that explore margin reduction [12]. Potentially, wide treatment margins, encompassing cortical tissue, may contribute to cerebral atrophy and late neurocognitive morbidity in patients who are long-term survivors.

Technological advances in neuroimaging, such as reduced slice thickness, three-dimensional reconstruction, and T2-FLAIR sequencing, have significantly improved initial tumour delineation. Non-enhancing tumour infiltration is increasingly recognised on MRI and amino acid positron emission tomography (PET) at sites distant from the primary contrast-enhancing lesion. In parallel, diffusion tensor imaging (DTI), already used clinically for tractography to guide neurosurgical procedures, has been explored in the context of the structural connectome, with some studies suggesting it may assist in detecting occult tumour invasion along white matter tracts [13].

This study aims to provide clinical data exploring the patterns of tumour infiltration and spread along anatomically connected brain regions by examining glioblastoma involving one region with low tumour incidence, the occipital lobe [14], with a focus on patterns of infiltration and subsequent tumour progression.

## 2. Methods

This retrospective study analysed consecutive adult patients (≥18 years) with newly diagnosed glioblastoma referred to the Neuro-Oncology Multidisciplinary Tumour Board between January 2008 and December 2023. Data for this study were stored in a prospectively maintained database approved by the Northern Sydney Local Health District Human Research Ethics Committee (reference LNR/15/HAWKE355).

Patients were eligible for inclusion if they had histologically confirmed glioblastoma (WHO Grade 4, isocitrate dehydrogenase (IDH) wildtype), involving the occipital lobe, and were managed according to the EORTC-NCIC protocol (adjuvant radiotherapy to 60 Gy with temozolomide). Exclusion criteria included patients with incomplete baseline or follow-up imaging and survival data.

Clinical data were extracted from the institutional electronic medical records and ethics-approved database, encompassing: (1) demographic characteristics; (2) tumour-specific parameters including histopathology, imaging characteristics, and O6-methylguanine-DNA methyltransferase (MGMT) promoter methylation status; (3) treatment details, including extent of surgical resection, radiation therapy dose/fractionation, and temozolomide use; (4) quantitative radiologic outcomes derived from diagnostic and progression MRI; and (5) survival endpoints (dates of progression and mortality).

### 2.1. Categorisation of Site of Infiltration and Progression

Three-dimensional tumour volumetric segmentation was performed manually using Eclipse™ treatment planning software 16.1 (Varian Medical Systems, Palo Alto, CA, USA). Volumetric assessments were conducted on both T1 gadolinium-enhanced MRI and T2-weighted FLAIR sequences at both preoperative diagnosis and at radiologically confirmed progression. Tumour infiltration was defined as either contrast-enhancing tumour on T1 gadolinium-enhanced MRI or suspicious T2-weighted FLAIR hyperintensity beyond the enhancing margin and was considered consistent with infiltrative tumour. For all measurements, residual tumour volume was delineated on MRI scans co-registered with computed tomography (CT) imaging to improve anatomical accuracy. Imaging assessments were based on review by an experienced specialist radiation oncologist with subspecialty expertise in neuro-oncology.

**Figure 1:**
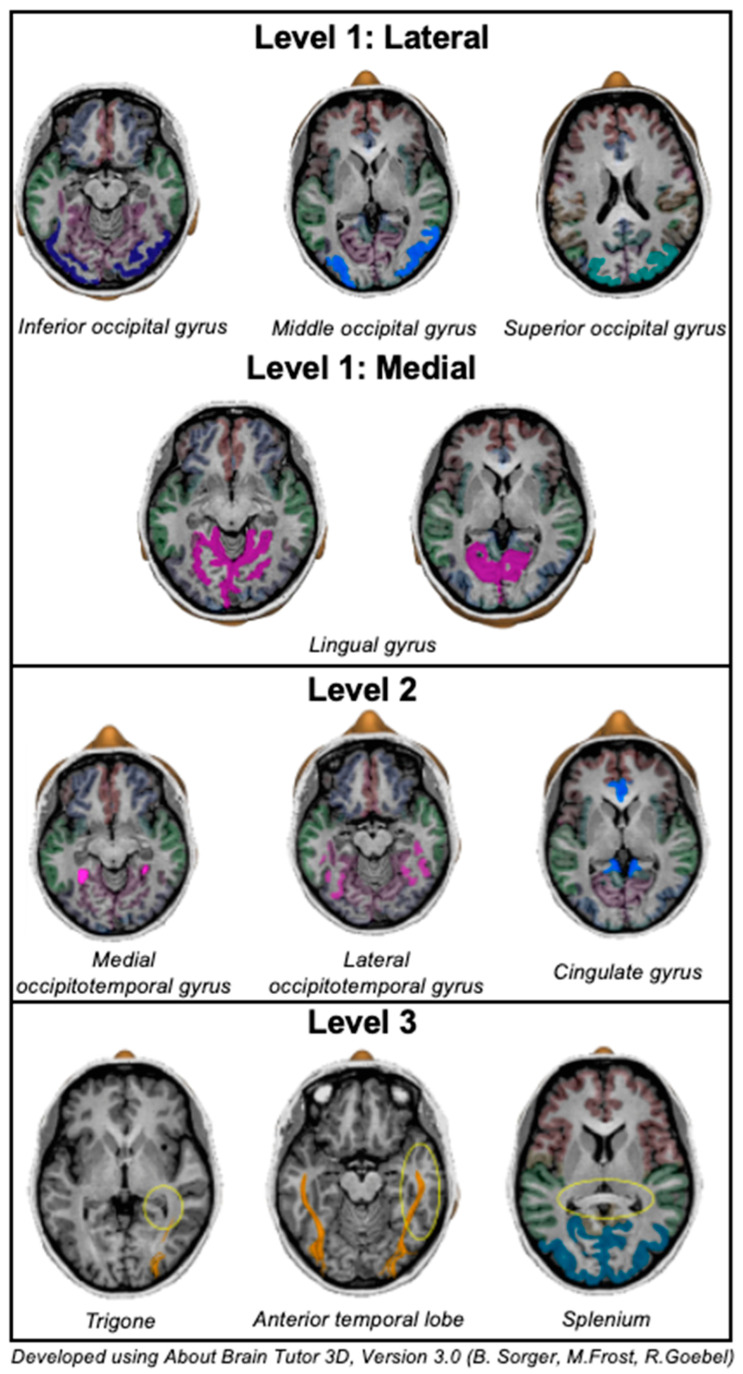
Neuroanatomical levels of infiltration.

These MRI volumes were then categorised into neuroanatomical subsites adjacent to the occipital lobe, designating three levels of infiltration (Figure 1):

• Level 1 origin subsites related to the involved gyrus: laterally, the inferior, middle, and superior occipital gyri; and medially, the lingual gyrus.
• Level 2 adjacent gyral subsites connected to the origin regions: medial occipitotemporal gyrus, lateral occipitotemporal gyrus, and cingulate gyrus.
• Level 3 distant subsites involving deeper extension: the trigone of the lateral ventricle, the anterior temporal lobe, and the splenium of the corpus callosum.

Each tumour was assigned a single level 1 origin based on its primary anatomical location at diagnosis. Extension to additional level 2 and/or level 3 subsites was permitted where multifocal or contiguous spread was identified at diagnosis or progression.

Spatial patterns were also assessed in relation to three adjacent major white matter tracts: inferior longitudinal fasciculus, cingulum, and corpus callosum.

### 2.2. Statistical Considerations

The primary endpoint involved a comprehensive qualitative description of sites of infiltration and progression and frequency of these sites in relation to the initial tumour subsite within the occipital lobe. These endpoints were analysed using descriptive statistics, and comparisons between groups were performed using Fisher’s exact test, given small sample sizes, with statistical significance defined as *p* < 0.05. Subsite-specific analyses were considered exploratory due to the modest cohort size and multiple anatomical comparisons performed; *p*-values are, therefore, reported descriptively and interpreted cautiously.

Secondary endpoints included median OS and progression-free survival (PFS), and the association with tumour subsite and established prognostic factors. Kaplan–Meier survival analysis was conducted, and Cox proportional hazards regression was used to evaluate the predictive value of measured variables. Given the exploratory nature of the survival analyses and the limited sample size, multivariable modelling was not performed to minimise overfitting. Statistical significance was defined as *p* < 0.05.

Statistical analyses were performed using Jamovi and IBM SPSS software (v28).

## 3. Results

A total of 46 patients with primary occipital lobe glioblastoma were managed during the study period and available for analysis. A total of 44 patients had died, with the two surviving patients progression-free at 21 and 45 months, respectively. Median OS was 17.4 months (95% CI: 15.1–19.9), and median PFS was 10.1 months (95% CI: 8.4–13.0). Patient characteristics are detailed and stratified by tumour origin in Table 1.

### 3.1. Infiltration Patterns

The defined anatomical levels were not mutually exclusive, and tumours could involve more than one level at diagnosis or progression. At diagnosis, tumours were localised to either the medial occipital lobe in 20 patients (43.5%) or the lateral occipital lobe in 26 patients (56.5%). Level 2 infiltration was observed in 33 patients (71.7%), and level 3 infiltration was observed in 27 patients (58.7%).

In medial tumours, 15 patients (75%) had level 2 and 11 patients (55%) had level 3 infiltration. In lateral tumours, 18 patients (69.2%) had level 2 and 16 patients (61.5%) had level 3 infiltration. There was no statistically significant difference in the presence of level 2 or 3 infiltration between medial and lateral subsites (*p* = 0.75 and *p* = 0.76, respectively).

Among patients with level 3 infiltration, medial tumours involved the trigone in seven patients (63.6%), the splenium in four patients (36.4%), and the anterior temporal lobe in one patient (9.1%). In lateral tumours, the trigone was involved in 15 patients (93.8%), the splenium in one patient (6.3%), and the anterior temporal lobe in three patients (18.8%). While trigone involvement was proportionally higher in lateral tumours and splenial involvement proportionally higher in medial tumours, these differences did not reach statistical significance (*p* = 0.15 for trigone, *p* = 0.15 for splenium, and *p* = 0.62 for anterior temporal lobe) (Table 2).

### 3.2. Progression Patterns

Progression occurred in 43 patients (93.5%), including 19 patients (95%) with medial tumours and 24 patients (92.3%) with lateral tumours.

Among patients with progression, involvement in medial tumours included level 1 in four patients (21.1%), level 2 in 13 patients (68.4%), and level 3 in 18 patients (94.7%). In lateral tumours, level 1 involvement was observed in eight patients (33.3%), level 2 in 20 patients (83.3%), and level 3 in 24 patients (100%). There was no statistically significant difference in level 3 involvement at progression based on initial tumour localisation (medial 18/19 patients (94.7%) vs. lateral 24/24 (100%) patients; *p* = 0.44).

**Figure 2:**
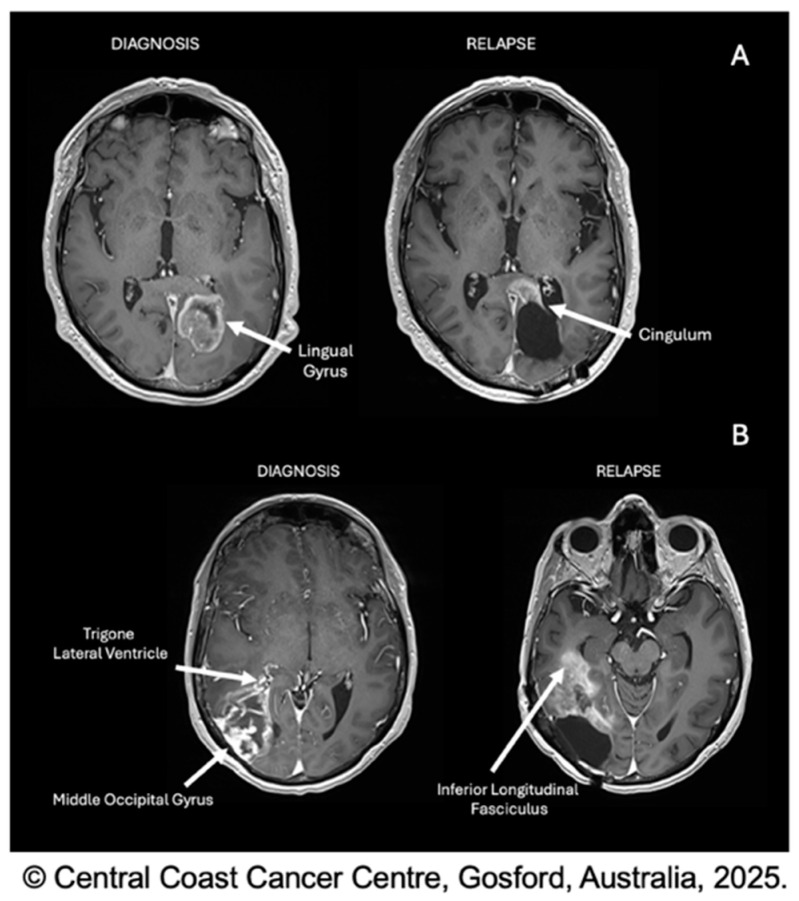
Axial T1-weighted gadolinium-enhanced MRI; examples of initial infiltration and progression patterns. (**A**) Medial tumour with progression involving the cingulum; (**B**) lateral tumour infiltrating towards the trigone, with progression involving the inferior longitudinal fasciculus.

At specific level 3 subsites, medial tumours involved the trigone in 10 patients (52.6%), the splenium in nine patients (47.3%), and the anterior temporal lobe in three patients (15.8%). Lateral tumours involved the trigone in 18 patients (75%), the splenium in four patients (16.7%), and the anterior temporal lobe in 12 patients (50%). Differences for the splenium (*p* = 0.046) and anterior temporal lobe (*p* = 0.026) reached nominal statistical significance, whereas the trigone did not (*p* = 0.198) (Table 3; Figure 2).

#### 3.2.1. Association of Occipital Lobe Subsite with Survival Outcome

Survival outcomes in relation to tumour localisation were not statistically significant.

Median OS was 16.3 months (95% CI: 13.1–24.0) for medial tumours and 18.0 months (95% CI: 15.1–23.6) for lateral tumours, with a hazard ratio (HR) of 0.95 (95% CI: 0.51–1.77; *p* = 0.876). Median PFS was 10.4 months (95% CI: 8.9–14.3) for medial tumours and 9.3 months (95% CI: 7.7–17.9) for lateral tumours, with an HR of 0.95 (95% CI: 0.52–1.75; *p* = 0.869; log-rank *p* = 0.87).

#### 3.2.2. Other Factors and Interaction with Survival Outcome

Higher ECOG scores at diagnosis tended to be associated with worse OS: 19.4 months for ECOG 0 (95% CI: 12.9–26.0), 17.1 months for ECOG 1 (95% CI: 14.7–19.6), and 11.4 months for ECOG 2 (95% CI: 11.0–11.7). Incremental increases in ECOG were significantly associated with worse OS (HR: 1.72; 95% CI: 1.14–2.58; *p* = 0.010). Although not statistically significant, progression-free survival (PFS) also tended to decline with increasing ECOG scores. Each one-point increase in ECOG was associated with a non-significant increase in progression risk (HR: 1.43; 95% CI: 0.96–2.13; *p* = 0.082).

MGMT promoter methylation status was available for 31 patients: 16 (51.6%) were unmethylated, and 15 (48.4%) were methylated. Median OS was longer in the methylated group at 23.6 months (95% CI: 15.7–31.6) compared to 14.3 months (95% CI: 10.8–17.7) in the unmethylated group. Methylation was associated with statistically significant improved OS (HR: 0.29; 95% CI: 0.12–0.69; *p* = 0.005). PFS was also longer in the methylated group, with a median of 17.9 months (95% CI: 8.0–27.8) versus 8.7 months (95% CI: 7.5–9.9) in the unmethylated group. The risk of progression was likewise improved and statistically significant in methylated tumours (HR: 0.18; 95% CI: 0.07–0.47; *p* < 0.001).

Higher Ki-67 expression was associated with poorer outcomes. Every 10% increase in Ki-67 corresponded to statistically significant shorter median OS (HR: 1.58; 95% CI: 1.28–1.96; *p* < 0.001) and shorter PFS (HR: 1.43; 95% CI: 1.17–1.76; *p* < 0.001).

With regard to the extent of surgical resection of enhancing tumour, classified according to the proportion of contrast-enhancing tumour removed on postoperative MRI as biopsy (<50% removed), partial resection (50–90% removed), and near-complete resection (>90% removed), there was no association with either OS or PFS. OS was 15.7 months (95% CI: 6.3–25.2) for biopsy, 15.5 months (95% CI: 8.3–22.6) for partial resection, and 18.1 months (95% CI: 14.0–22.3) for near-complete resection. There was no statistically significant difference in OS when biopsy was compared to partial resection (HR: 0.72; 95% CI: 0.21–2.47; *p* = 0.597) or near-complete resection (HR: 0.58; 95% CI: 0.17–1.95; *p* = 0.376). Median PFS was 13.1 months (95% CI: 4.1–22.1) for biopsy, 10.2 months (95% CI: 9.3–11.2) for partial resection, and 8.9 months (95% CI: 5.9–11.8) for complete resection. As with OS, there was no statistically significant difference in PFS when comparing partial resection (HR: 1.03; 95% CI: 0.30–3.52; *p* = 0.966) or near-complete resection (HR: 0.80; 95% CI: 0.23–2.73; *p* = 0.721) to biopsy.

## 4. Discussion

This study provides a focused analysis of occipital lobe glioblastoma, a relatively uncommon tumour location [14], and examines the patterns of initial infiltration, progression, and survival outcomes according to tumour subsite within the occipital lobe.

Although the cohort is modest in size, there was a reasonable distribution of medial (20 patients) and lateral (26 patients) occipital tumours. Tumour infiltration at diagnosis was common beyond the occipital lobe in both medial and lateral occipital subsites. Over 70% of patients demonstrated level 2 infiltration, and 58.7% demonstrated level 3 infiltration.

Interestingly, analysis of progression patterns suggested that progression in this cohort of occipital glioblastoma frequently involved distant anatomical regions rather than remaining confined to the original tumour location. High rates of recurrence were observed at level 2 and particularly at level 3, regardless of whether the tumour originated at a medial or lateral subsite within the occipital lobe. Only 21.1% of medial and 33.3% of lateral tumours progressed at level 1. In contrast, level 2 involvement at progression was seen in 68.4% of medial tumours and 83.3% of lateral tumours, while level 3 involvement occurred in nearly all cases (94.7% and 100%, respectively). These findings suggest that, within this cohort, local or marginal tumour progression was relatively uncommon. It should be noted that level 3 encompasses several distinct anatomical subsites, which may increase the probability of detecting progression within this category.

There was no statistically significant difference in survival outcomes according to the extent of surgery. Within the limits of sample size, this suggests that the high risk of distant infiltration may be more prominent than local disease. It may support the hypothesis that progression occurs in predictable pathways rather than isotropic extension from the original contrast-enhancing mass.

**Figure 3:**
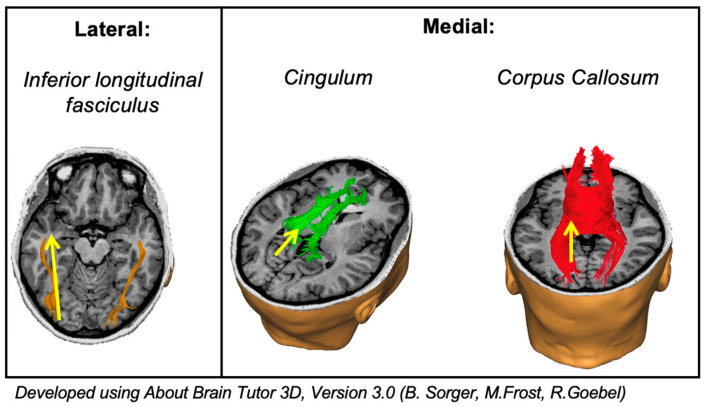
Schematic illustration of major white matter tracts within the vicinity of the medial and lateral occipital lobes and potential direction and routes for tumour infiltration. The lateral occipital lobe potential route of infiltration along the inferior longitudinal fasciculus towards the ventricular trigone and the anterior temporal lobe. The medial occipital lobe potential route of infiltration along the cingulum and corpus callosum to the splenium.

Between occipital subsites, differences were observed in progression distribution at specific level 3 subsites, with some reaching nominal statistical significance. Medial tumours more frequently involved the splenium and less frequently the anterior temporal lobe compared with lateral tumours. Although not statistically significant, trigone involvement seemed to be more prominent amongst lateral tumours (lateral 75% vs. medial 52.6%). Although the findings on analysis of infiltration were not statistically significant, they correlated with progression patterns where splenium involvement appeared more common in medial tumours and trigone involvement more common in lateral tumours. Interpreted cautiously, given small sample sizes, these findings are anatomically compatible with potential subsite-specific routes of tumour progression and may reflect differences in white matter connectivity. Medially, the major white matter tracts, such as the cingulum and the corpus callosum, emerge from this region and thus extend into the splenium. Laterally, the inferior longitudinal fasciculus extends towards the trigone and anterior temporal lobe (Figure 2 and Figure 3).

The OS of 17.4 months and PFS of 10.1 months are in keeping with reported survival outcomes for glioblastoma [3,4,5]. This suggests that occipital localisation of glioblastoma may not have a substantially different prognosis compared to tumours in other brain regions. Despite the anatomical distinctions in progression patterns mentioned above, no statistically significant survival difference between medial and lateral occipital tumours was observed.

The limitations of this study include its retrospective design and undoubtedly its relatively small sample size. Additionally, subgroup analyses were limited, and certain variables, such as MGMT promoter methylation status, were not available for all patients. Furthermore, given the relative rarity of occipital lobe glioblastoma, the comparative literature is limited, which restricts broader contextualisation of our findings.

## 5. Conclusions

This study contributes valuable insight into the behaviour of occipital lobe glioblastoma. The high frequency of level 3 progression involving deep structures such as the trigone, the splenium, and the anterior temporal lobe suggests that traditional isotropic margin expansion in radiotherapy based solely on the initial tumour location may underestimate the true extent of disease. These findings should be considered hypothesis-generating and support further investigation into whether incorporating likely anatomical pathways of spread, particularly white matter tracts, could inform future radiotherapy planning.

## Figures and Tables

**Table 1:** Baseline characteristics according to tumour origin.

Characteristic	Medial (*n* = 20)	Lateral (*n* = 26)	Overall (*n* = 46)
Median age at diagnosis (IQR)	62.0 (51.2–71.5)	60.4 (51.8–64.6)	60.7 (51.4–65.7)
Initial ECOG ^a^ performance status			
0	6 (30.0%)	7 (26.9%)	13 (28.2%)
1	7 (35.0%)	18 (69.2%)	25 (54.3%)
2	6 (30.0%)	1 (3.8%)	7 (15.2%)
3	1 (5.0%)	0 (0%)	1 (2.2%)
4	0 (0%)	0 (0%)	0 (0%)
MGMT ^b^ methylation status			
Unmethylated	8 (40.0%)	8 (30.8%)	16 (34.7%)
Methylated	6 (30.0%)	9 (34.6%)	15 (32.6%)
Unknown	6 (30.0%)	9 (34.6%)	15 (32.6%)
Ki-67 proliferation index			
Median Ki-67 (IQR)	30% (22.5–42.5)	30% (25–45)	30% (23.8–45.0)
0–25%	5 (25.0%)	9 (34.6%)	14 (30.4%)
26–50%	7 (35.0%)	14 (53.8%)	21 (45.6%)
51–75%	2 (10.0%)	1 (3.8%)	3 (6.5%)
76–100%	1 (5.0%)	1 (3.8%)	2 (4.3%)
Unavailable	5 (25.0%)	1 (3.8%)	6 (13.0%)
Extent of resection			
Biopsy	2 (10.0%)	1 (3.8%)	3 (6.5%)
Subtotal resection	7 (35.0%)	11 (42.3%)	18 (39.1%)
Gross total resection	11 (55.0%)	14 (53.8%)	25 (54.3%)

Continuous variables are presented as median (interquartile range [IQR]); categorical variables as number (percentage). Percentages are calculated within each tumour origin group and for the overall cohort. ^a^ ECOG, Eastern Cooperative Oncology Group; ^b^ MGMT, O6-methylguanine-DNA methyltransferase.

**Table 2:** Infiltration patterns, comparing medial and lateral (level 1) origin of the occipital lobe glioblastoma.

Variable	Medial (*n* = 20)	Lateral (*n* = 26)	Total *n*	*p*-Value
Level 2 infiltration	15 (75.0%)	18 (69.2%)	33 (71.7%)	0.75
Level 3 infiltration	11 (55.0%)	16 (61.5%)	27 (58.7%)	0.76
Level 3 subsite infiltration *				
Trigone	7 (63.6%)	15 (93.8%)	22 (81.4%)	0.15
Splenium	4 (36.4%)	1 (6.3%)	5 (18.5%)	0.15
Anterior temporal lobe	1 (9.1%)	3 (18.8%)	4 (14.8%)	0.62

Percentages for level 2 and level 3 infiltration are calculated using the total number of medial (*n* = 20) and lateral (*n* = 26) tumours as denominators. * Percentages for level 3 subsite involvement are calculated using only patients with level 3 infiltration (medial *n* = 11; lateral *n* = 16). Subsite categories are not mutually exclusive. *p*-values correspond to Fisher’s exact test comparisons between medial and lateral tumour origin.

**Table 3:** Progression patterns, comparing medial and lateral (level 1) origin of occipital lobe glioblastoma.

Variable	Medial (*n* = 19)	Lateral (*n* = 24)	Total (*n* = 43)	*p*-Value
Level 1 involvement	4 (21.1%)	8 (33.3%)	12 (27.9%)	-
Level 2 involvement	13 (68.4%)	20 (83.3%)	33 (76.7%)	0.295
Level 3 involvement	18 (94.7%)	24 (100%)	42 (97.7%)	0.440
Level 3 subsite infiltration				
Trigone	10 (52.6%)	18 (75.0%)	28 (66.7%)	0.198
Splenium	9 (47.3%)	4 (16.7%)	13 (31.0%)	0.046
Anterior temporal lobe	3 (15.8%)	12 (50%)	15 (35.7%)	0.026

Percentages are calculated using the number of patients with progression in each tumour origin group (medial *n* = 19; lateral *n* = 24; total *n* = 43). Subsite categories are not mutually exclusive; tumours may involve more than one anatomical level or subsite at progression. *p*-values correspond to Fisher’s exact test comparisons between medial and lateral tumour origin.

## Data Availability

The datasets generated and/or analysed during the current study are not publicly available due to patient privacy and ethical restrictions related to the use of a prospectively maintained clinical database. De-identified data may be made available from the corresponding author upon reasonable request and subject to institutional ethics approval.
